# Supplementary material for: Infant Mode of Delivery Shapes the Skin Mycobiome of Prepubescent Children
Source: Microbiol Spectr. 2022 Sep 8;10(5):e02267-22. doi: 10.1128/spectrum.02267-22 (PMC9603757; doi:10.1128/spectrum.02267-22)
Supplement: Supplemental file 1 — Tables S1 to S5 and Fig. S1 to S8. Download spectrum.02267-22-s0001.pdf, PDF file, 2.3 MB [file spectrum.02267-22-s0001.pdf]

Supplemental Material FOR Publication

Supplemental Tables 2

Table S1 Demographics of participant children and mothers 2

Table S2 Mantel test of Bray-Curtis distance matrices and skin physical parameters. 3

Table S3 Pearson's correlation analysis of the relative abundances of main fungal genera and physical skin parameters 4

Table S4 Pearson's correlation analysis between the relative abundances of fungal genera with children's age. 5

Table S5 Spearson's correlation analysis between mothers and their children for different age groups, modes of delivery and skin sites 6

Supplemental Figures 8

Fig. S1 PCoA analysis for main factors affecting skin mycobiomes in children 8

Fig. S2 Sloan neutral model predictions of cutaneous fungi and bacteria 9

Fig. S3 Comparison of alpha diversity among children of different delivery modes 10

Fig. S4 General changes in Bray-Curtis dissimilarity trajectories across children's ages 11

Fig. S5 Major fungal genera in one-year-old children for different modes of delivery. 12

Fig. S6 Spearman's correlation test of several major fungal genera between mothers and their children for different modes of delivery and age groups 13

Fig. S7 Bray-Curtis dissimilarity trajectory of the skin mycobiome across children's ages based on microbiome data without filtering and rarefaction 14

Fig. S8 Bray-Curtis dissimilarity trajectory of the skin mycobiome across children's ages based on samples from children aged 1, 2, 3 and 5 15

Supplemental Tables

Table S1 Demographics of participant children and mothers[a].

Healthy children analyzed (n=number of participant children)	Age	Sex	Delivery mode	Birthplace	Feeding type
	1 : 2 : 3 : 4 : 5 : 10	M : F	C-section : Vaginal	Ub : Sb	Br : Fm : Mi
**all children, n=72**	13:10:12:14:13:10	30:42	28:44	29:43	28:16:28
**1-year-old, n=13**	—	4:9	5:8	10:3	1:4:8
**2-year-old, n=10**	—	5:5	6:4	7:3	3:1:6
**3-year-old, n=12**	—	5:7	4:8	4:8	3:4:5
**4-year-old, n=14**	—	4:10	3:11	1:13	11:1:2
**5-year-old, n=13**	—	8:5	8:5	3:10	5:3:5
**10-year-old, n=10**	—	4:6	2:8	4:6	5:3:2
**C-section, n=28**	5:6:4:3:8:2	14:14	—	14:14	10:8:10
**Vaginal, n=44**	8:4:8:11:5:8	16:28	—	15:29	18:8:18
**Ub, n=29**	10:7:4:1:3:4	10:19	14:15	—	7:7:15
**Sb, n=43**	3:3:8:13:10:6	20:23	14:29	—	21:9:13

[a]Abbreviations: Numbers (1, 2, 3, 4, 5, and 10) represent children's ages. M: male, F: female, Ub: urban, Sb: suburban, Br: breastfed, Fm: formula-fed, Mi: Mix-fed.

Table S2 Mantel test of Bray-Curtis distance matrices and skin physical parameters.

Groups[a,b]	Moisture		TEWL		pH	
	R[d]	p_{fdr}	R[d]	p_{fdr}	R[d]	p_{fdr}
**1**[c]	0.1899	0.216	0.3551**	0.006	0.0736	0.456
**2**[c]	–0.1839	0.128	–0.2422	0.105	–0.0696	0.368
**3**[c]	0.1897	0.090	–0.0524	0.535	0.0073	0.951
**4**[c]	–0.1395	0.130	0.0559	0.389	–0.0303	0.720
**5**[c]	0.1748	0.182	0.1534	0.193	0.1062	0.303
**10**[b]	0.0613	0.573	–0.1384	0.174	–0.0929	0.356
**1.Fa**	0.3712	0.106	0.5510**	0.005	–0.0044	0.984
**1.Vf**	–0.0439	0.808	0.2200	0.250	–0.0655	0.677
**2.Fa**	–0.1948	0.432	–0.1989	0.336	0.1507	0.411
**2.Vf**	–0.0638	0.734	0.0577	0.821	0.0790	0.713
**3.Fa**	0.4830*	0.011	0.0421	0.783	0.0008	0.999
**3.Vf**	–0.1692	0.184	–0.1734	0.234	0.0240	0.896
**4.Fa**	–0.0959	0.644	–0.0596	0.753	0.2110	0.241
**4.Vf**	0.0434	0.766	0.2813	0.062	–0.2251*	0.050
**5.Fa**	0.0705	0.755	0.1086	0.591	0.5387**	0.006
**5.Vf**	0.1595	0.133	–0.2194	0.183	0.1578	0.329
**10.Fa**	0.2189	0.314	–0.3521	0.102	–0.0952	0.592
**10.Vf**	–0.2235	0.218	–0.2471	0.195	–0.0075	0.958

[a]Numbers (1, 2, 3, 4, 5, and 10) represent children's ages.

[b]Abbreviations: Fa: Face, Vf: Ventral forearm.

[c]Including both sites (face and ventral forearm).

[d]*: $p_{fdr} \leq 0.05$, **: $p_{fdr} \leq 0.01$.

Table S3 Pearson's correlation analysis of the relative abundances of main fungal genera and physical skin parameters[a].

Genus	TEWL	Moisture			pH
	children	3[b]	3.Fa	10.Vf	5.Fa
	R[d]	R[d]	R[d]	R[d]	R[d]
Malassezia	0.0670	–0.0723	–0.3201	–0.8581*	–0.8294**
Cladosporium	–0.1249	0.4776	0.6784	0.0430	0.0161
Alternaria	0.0068	–0.1628	–0.1583	–0.0540	0.1290
Candida	0.0460	–0.0586	–0.2077	–0.5272	–0.2346
Rhodotorula	0.0062	–0.0792	–0.1057	0.5405	0.3660
Aspergillus	–0.1056	–0.1491	–0.3268	–0.5361	0.3693
Peyronellaea	–0.1726	–0.1769	–0.2608	0.0528	0.3936
Sterigmatomyces	0.1332	0.8415***	0.9429***	–0.0026	0.2946
Cryptococcus	–0.0738	–0.2205	–0.2841	–0.1386	–0.0875
Aureobasidium	–0.0427	–0.1093	–0.1522	–0.0680	0.4199
Fusarium	–0.0341	–0.0610	–0.1590	–0.1153	0.1285
Sporobolomyces	0.2830**	–0.1720	–0.1933	0.0460	0.3569
Debaryomyces	–0.0629	0.1142	0.1291	0.4034	–0.0187
Cystofilobasidium	0.0306	–0.0314	–0.1544	0.0728	–0.0431
Meyerozyma	0.0712	–0.1242	–0.1070	0.4351	0.3273

[a]Numbers (3 and 5) represent children's ages.

[b]Including both sites (face and ventral forearm).

[c]Abbreviations: Fa: Face, Vf: Ventral forearm.

[d]*: $p_{fdr} \leq 0.05$, **: $p_{fdr} \leq 0.01$, ***: $p_{fdr} \leq 0.0001$.

Table S4 Pearson's correlation analysis between the relative abundances of fungal genera with children's ages.

Genus[a]	Vaginally-born		Caesarean-born	
	R	P_{fdr}	R	P_{fdr}
Malassezia	-0.2191	0.1432	-0.2486	0.3843
Cladosporium	-0.0583	0.8505	0.2427	0.3945
Alternaria	0.2092	0.1683	-0.0633	0.9206
Candida	-0.2248	0.1432	-0.0971	0.9206
Rhodotorula	-0.1738	0.2863	-0.1855	0.9206
Aspergillus	0.0378	0.9284	0.0007	0.9952
Peyronellaea	0.0599	0.8505	0.0414	0.9206
Sterigmatomyces	-0.0534	0.8898	0.0244	0.9206
Cryptococcus	-0.0714	0.8505	0.2040	0.7758
Aureobasidium	0.0660	0.8505	-0.0247	0.9206
Fusarium	0.3993	0.0003	0.3291	0.0964
Sporobolomyces	0.0214	0.9558	-0.0975	0.9206
Debaryomyces	-0.0773	0.8492	0.0209	0.9275
Cystofilobasidium	-0.1902	0.2579	0.3059	0.0964
Meyerozyma	-0.0100	0.9687	-0.1107	0.9206

[a]Results of the main fungal genera (mean relative abundance > 1%) in samples from children are shown. Multiple Pearson's correlation tests of all fungal genera in children are performed to adjust the *P*-value using the false discovery rate method.

Table S5 Spearman's correlation analysis between mothers and their children for different age groups, modes of delivery, and skin sites[a].

Genus[b]	Group aged from 1-3 years old (n = 105)												Group aged from 4-5, 10 years old (n = 111)											
	Vaginally-born (n = 60)						Caesarean-born (n = 45)						Vaginally-born (n = 72)						Caesarean-born (n = 39)					
	Face (n = 20)		Ventral forearm (n = 20)		Calf (n = 20)		Face (n = 15)		Ventral forearm (n = 15)		Calf (n = 15)		Face (n = 24)		Ventral forearm (n = 24)		Calf (n = 24)		Face (n = 13)		Ventral forearm (n = 13)		Calf (n = 13)	
	Rho[c]	P_{fdr}	Rho[c]	P_{fdr}	Rho[c]	P_{fdr}	Rho[c]	P_{fdr}	Rho[c]	P_{fdr}	Rho[c]	P_{fdr}	Rho[c]	P_{fdr}	Rho[c]	P_{fdr}	Rho[c]	P_{fdr}	Rho[c]	P_{fdr}	Rho[c]	P_{fdr}	Rho[c]	P_{fdr}
Malassezia	0.1910	0.7894	0.1414	0.8282	0.3368	0.5549	0.2520	0.6778	0.1536	0.8463	0.1571	0.9177	0.1840	0.6892	-0.0930	0.8444	0.2121	0.7802	0.4176	0.6108	0.5769	0.2172	0.5989	0.2408
Cladosporium	0.4212	0.2489	-0.0135	0.9746	-0.0571	0.8840	0.2893	0.6387	0.6077	0.2392	0.1646	0.9177	0.5145	0.1031	0.4017	0.2226	-0.1042	0.9054	0.4725	0.4459	0.4033	0.4260	0.4615	0.3749
Alternaria	-0.0192	0.9456	0.2988	0.4742	0.2094	0.7830	0.3318	0.5887	0.1843	0.8463	-0.0984	0.9224	0.3465	0.3584	-0.1980	0.6623	0.0116	0.9572	0.3411	0.6490	0.7088	0.0823	0.4780	0.3487
Candida	0.3504	0.4197	0.2408	0.6183	0.5520	0.1205	0.3467	0.5734	-0.0447	0.9284	-0.2601	0.9177	0.0957	0.8659	-0.1315	0.8203	0.2495	0.7328	0.2613	0.6850	0.4615	0.3982	0.1157	0.8396
Rhodotorula	0.2821	0.5754	0.4827	0.1633	0.3635	0.4948	0.2272	0.7170	-0.1162	0.8512	0.0680	0.9224	-0.0788	0.8659	0.3237	0.3538	0.1125	0.9051	-0.1348	0.8385	-0.2167	0.7857	0.4869	0.3487
Aspergillus	0.4447	0.2186	0.4831	0.1633	0.2434	0.7365	0.4614	0.4558	0.4395	0.4170	0.5556	0.5424	0.1054	0.8659	-0.1783	0.7157	0.2456	0.7328	0.1657	0.8272	0.3132	0.6188	0.2088	0.8170
Fusarium	0.2005	0.7894	0.2175	0.6654	0.1529	0.8724	0.0940	0.8409	0.0117	0.9845	-0.0913	0.9224	0.6742	0.0070	0.3640	0.2576	0.5861	0.0414	0.3686	0.6490	0.4014	0.4260	0.6352	0.1643
Peyronellaea	0.2430	0.6484	0.3227	0.4543	0.0306	0.9400	-0.0977	0.8393	0.2057	0.8463	0.3703	0.8741	-0.2607	0.5238	0.5290	0.0868	0.0543	0.9475	-0.4780	0.4459	0.6000	0.1721	0.4377	0.3756
Sterigmatomyces	0.5210	0.1137	-0.0842	0.9132	-0.0916	0.8724	0.2250	0.7170	0.4541	0.3987	0.3634	0.8741	-0.0704	0.8659	-0.1776	0.7157	-0.0949	0.9298	-0.0999	0.8385	-0.2863	0.6772	-0.5980	0.2366
Aureobasidium	0.1827	0.8063	0.2748	0.5299	0.0526	0.8840	-0.4525	0.4558	0.1543	0.8463	0.2813	0.9147	-0.0571	0.8664	0.0688	0.8498	0.1330	0.9007	-0.2216	0.7547	0.1312	0.8478	0.4551	0.3749
Cryptococcus	0.2732	0.5850	0.2129	0.6735	-0.0257	0.9410	0.2572	0.6778	0.2786	0.8081	-0.2154	0.9177	0.3638	0.3431	0.0081	0.9821	-0.0378	0.9514	0.2672	0.6783	0.3609	0.5213	-0.0157	0.9594
Sporobolomyces	-0.3139	0.4794	-0.0642	0.9193	0.6125	0.0520	0.3285	0.5887	-0.0936	0.8742	-0.0371	0.9719	0.3419	0.3584	0.1212	0.8203	0.4780	0.1339	0.2424	0.7227	-0.5710	0.2172	0.6849	0.1001
Penicillium	0.1966	0.7894	0.1914	0.7220	0.3462	0.5294	-0.2728	0.6571	0.1655	0.8463	0.1845	0.9177	0.0772	0.8659	0.3288	0.3532	0.1841	0.8308	0.2741	0.6783	0.4380	0.4260	0.3344	0.6396
Debaryomyces	0.1519	0.8498	0.5749	0.0684	-0.0171	0.9611	0.4533	0.4558	-0.0830	0.8796	-0.1842	0.9177	0.2744	0.4975	0.3887	0.2398	0.0650	0.9298	0.4965	0.4412	0.0855	0.8478	0.4986	0.3487
Cystofilobasidium	-0.0188	0.9456	0.6047	0.0650	0.2412	0.7365	0.3095	0.6387	0.5453	0.2680	-0.1395	0.9177	0.2010	0.6750	0.5224	0.0868	0.2363	0.7328	0.4851	0.4459	0.2380	0.7727	0.1478	0.8396
Meyerozyma	0.4470	0.2186	0.5870	0.0650	0.6516	0.0393	0.2917	0.6387	0.1475	0.8463	0.1700	0.9177	0.4742	0.1399	0.4828	0.1327	0.0865	0.9298	0.3865	0.6490	0.4047	0.4260	0.1746	0.8170

[a]n indicates number of pairs of mothers and their children.

[b]Results of the main fungal genera (mean relative abundance > 1%) in samples of mothers and children are shown. Multiple Spearman's correlation tests of all fungal genera in children and mothers are performed to adjust the *P*-value using the false discovery rate method.

[c]Rho-values represent the strength of the Spearman's correlation.

Supplemental Figures

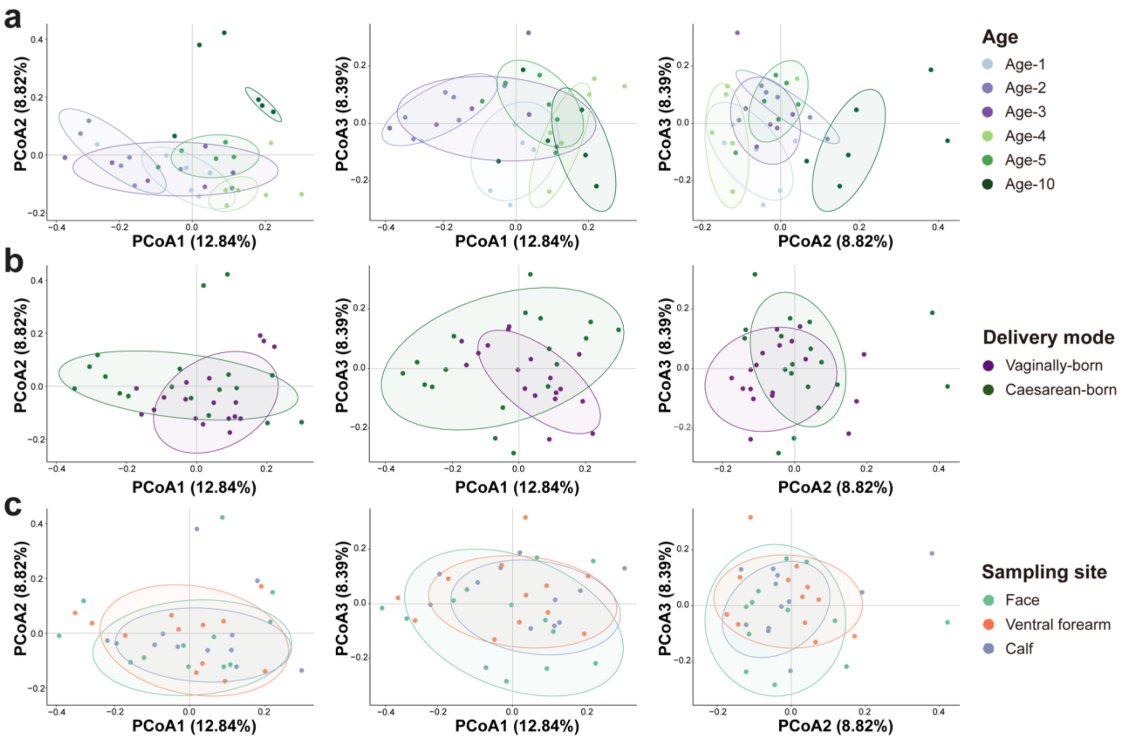

Fig. S1 PCoA analysis for the main factors affecting skin mycobiomes in children. (a–c) PCoA analysis of axes 1 vs. 2, 1 vs. 3, and 2 vs. 3 based on the Bray-Curtis dissimilarity of the fungal community towards the main factors (i.e., for (a) children's ages, (b) delivery mode, and (c) sampling sites) for 72 groups of samples. The samples are grouped based on the children's ages, delivery mode, and sampling site.

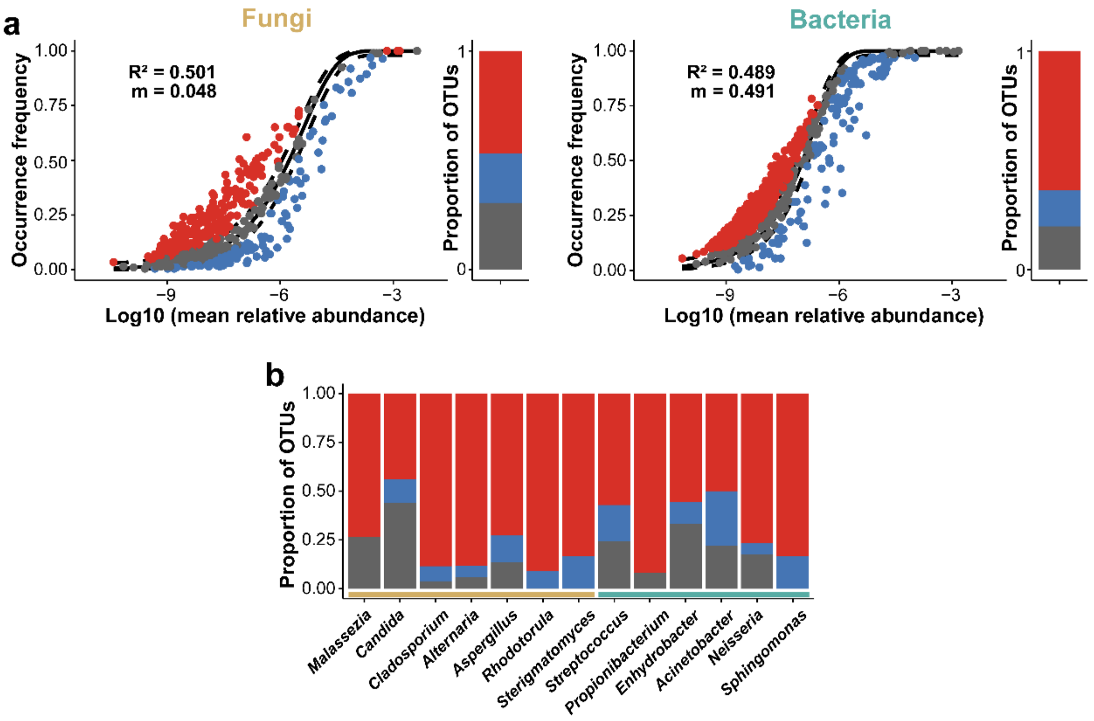

Fig. S2 Sloan neutral model predictions of cutaneous fungi and bacteria. (a) Sloan neutral model prediction of cutaneous fungi and bacteria. Data points represent the OTUs and the colors represent whether the OTUs fitted above (red), within (grey), or below (blue) the 95% confidence interval (dotted lines) of the neutral model prediction. R^2 values (fitness to neutral assembly process) and m values (estimated migration rate) are shown. (b) Proportions of specific OTUs of the core cutaneous fungal or bacterial genera (mean relative abundance > 3%) according to the Sloan neutral model prediction.

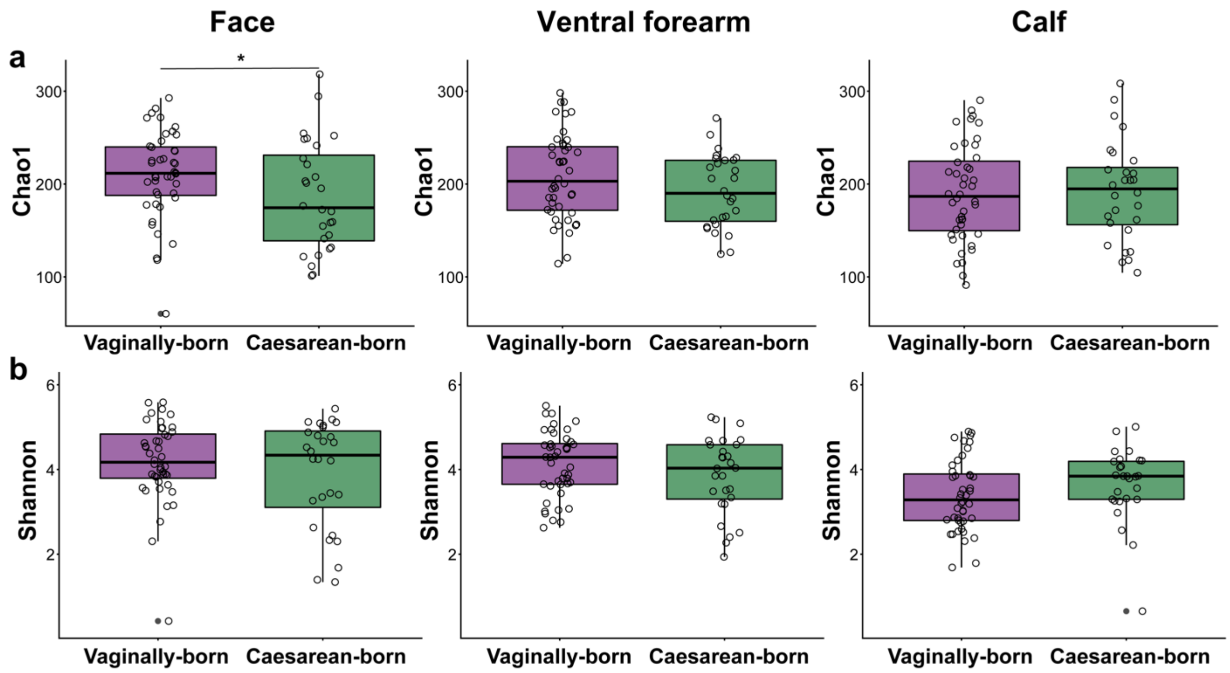

Fig. S3 Comparison of alpha diversity among children of different delivery modes. (a–b) Comparison of alpha diversity ((a) for Chao1, evaluated as richness, (b) for Shannon, evaluated as evenness) among children of different delivery modes in three sampling sites. Data points represent alpha diversity indices, containing skin samples of vaginally-born (purple) and caesarean-born (green) children. *: $P \leq 0.05$ based on Wilcoxon tests.

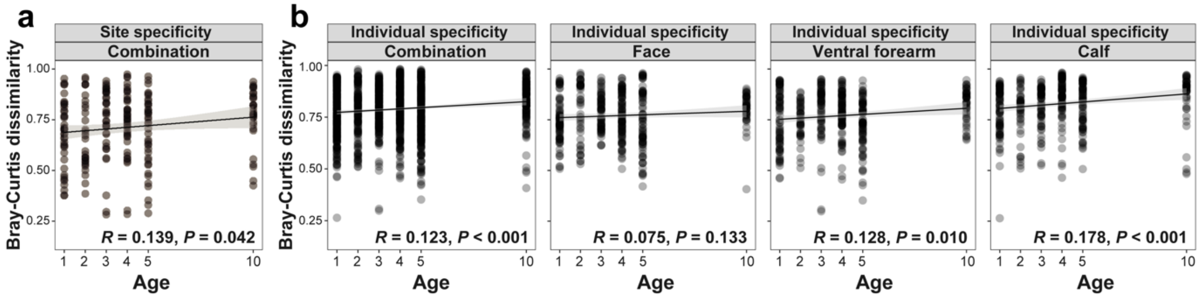

Fig. S4 General changes in Bray-Curtis dissimilarity trajectories across children's ages. (a) Site specificity as evaluated by the Bray-Curtis dissimilarity of mycobiomes within different skin sites (intra-individual dissimilarity). R and *P*-values of the Pearson's correlation test are shown. (b) Individual specificity as evaluated by the Bray-Curtis dissimilarity of mycobiomes within different individuals (inter-individual dissimilarity) of different sampling sites.

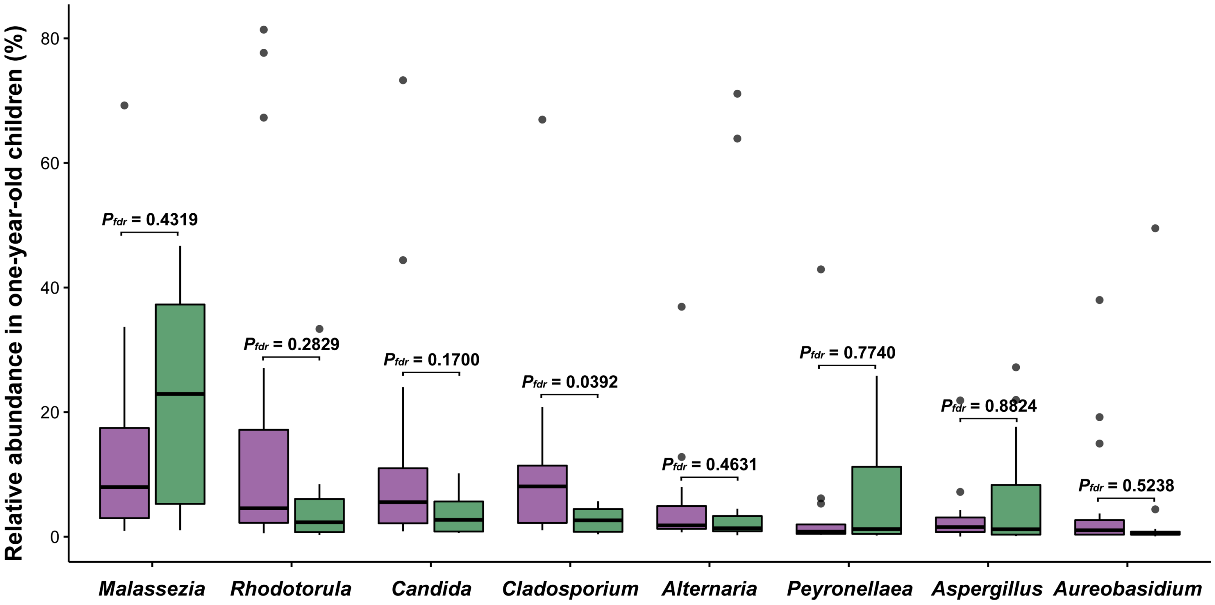

Fig. S5 Major fungal genera in one-year-old children for different modes of delivery. Top abundant fungal genera (mean relative abundance ≥ 3%) within one-year-old children of different delivery modes (n = 8 for the vaginally-born, n = 5 for the caesarean-born). Multiple Wilcoxon tests of all fungal genera in children are performed to adjust the *P*-value using the false discovery rate method. P_{fdr}-values of the Wilcoxon test are presented. Colors represent skin samples of vaginally-born (purple) and caesarean-born (green) children.

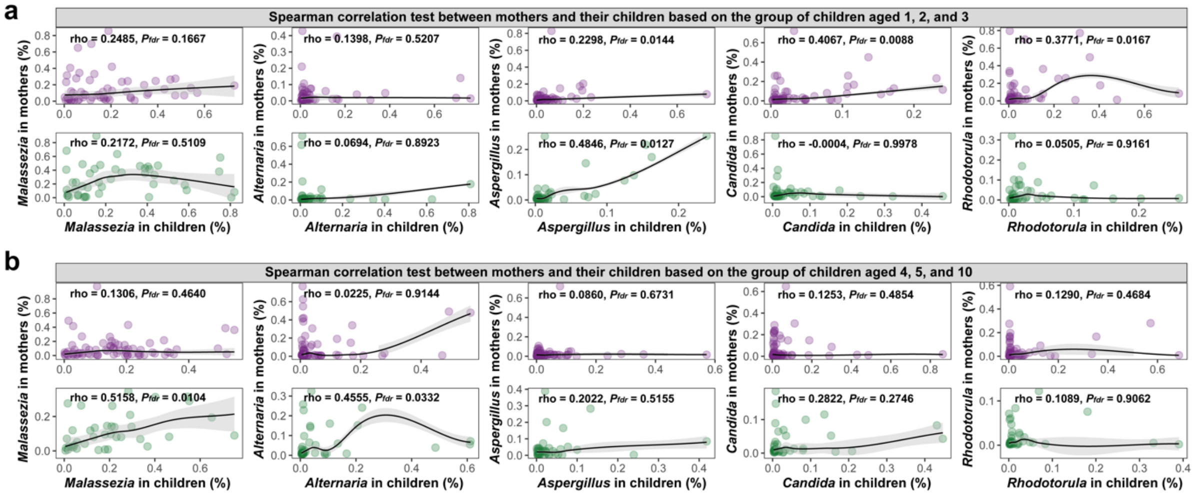

Fig. S6 Spearman's correlation test of several major fungal genera between mothers and their children for different modes of delivery and age groups (a: younger group from children aged 1, 2, and 3, b: elder group from children aged 4, 5, and 10). Multiple Spearman's correlation tests of all fungal genera in children and mothers are performed to adjust the *P*-value using the false discovery rate method. Points represent the relative abundances from mothers and their children, containing skin samples of vaginally-born (purple) and caesarean-born (green) children. Rho- and P_{fdr}-values of the Spearman's correlation test are shown.

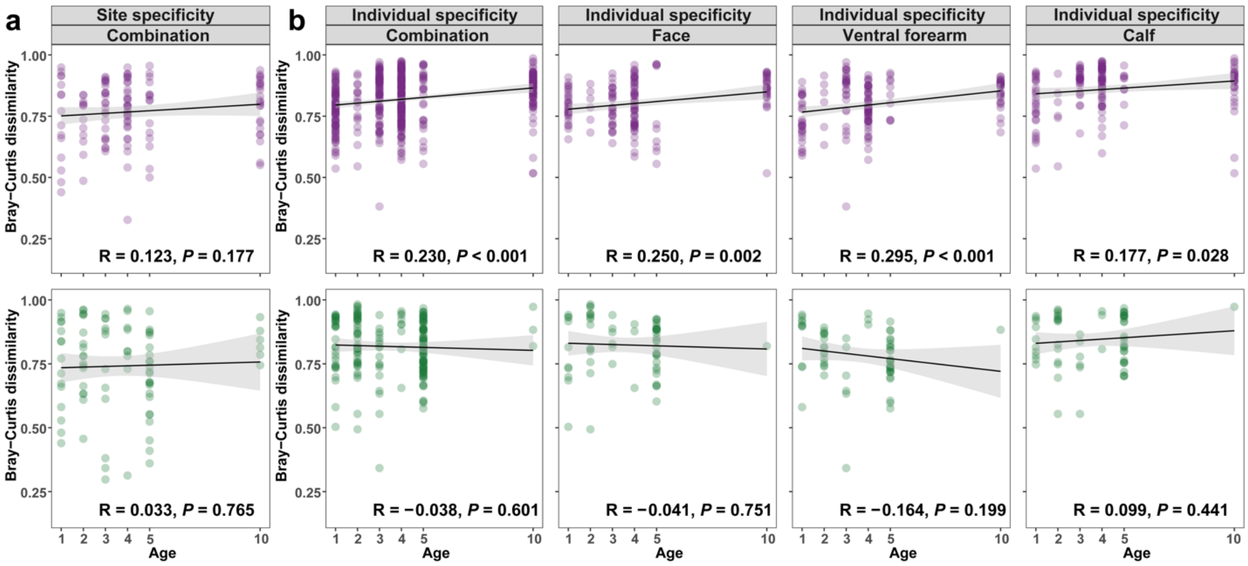

Fig. S7 Bray-Curtis dissimilarity trajectory of the skin mycobiome across children's ages based on microbiome data without filtering and rarefaction. (a) Site specificity as evaluated by the Bray-Curtis dissimilarity of the mycobiomes within different skin sites (intra-individual dissimilarity) from vaginally-born and caesarean-born children. (b) Individual specificity as evaluated by the Bray-Curtis dissimilarity of the mycobiomes within different individuals (inter-individual dissimilarity) of different skin sites in vaginally-born and caesarean-born children. Points represent the Bray-Curtis dissimilarity indices containing skin samples of vaginally-born (purple) and caesarean-born (green) children. R and *P*-values of the Pearson's correlation test are shown.

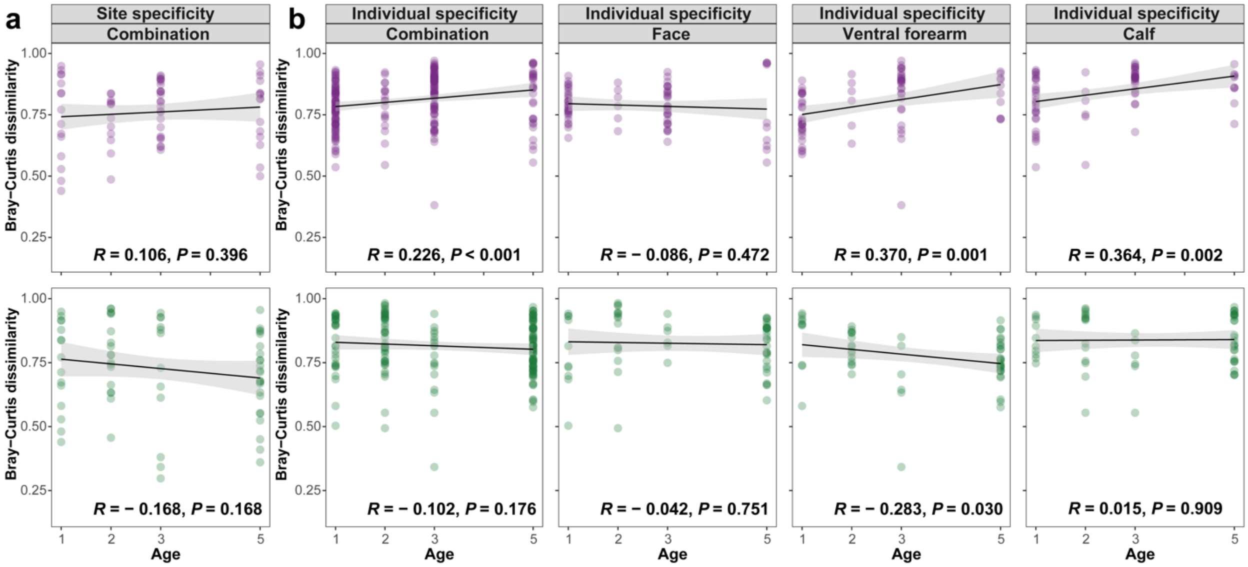

Fig. S8 Bray-Curtis dissimilarity trajectory of the skin mycobiomes across children's ages based on samples from children aged 1, 2, 3, and 5. (a) Site specificity as evaluated by Bray-Curtis dissimilarity of the mycobiomes within different skin sites (intra-individual dissimilarity) from vaginally-born and caesarean-born children. (b) Individual specificity as evaluated by the Bray-Curtis dissimilarity of the mycobiome within different individuals (inter-individual dissimilarity) of different skin sites in vaginally-born and caesarean-born children. Points represent the Bray-Curtis dissimilarity indices containing skin samples of vaginally-born (purple) and caesarean-born (green) children. R and *P*-values of the Pearson's correlation test are shown.
